# Effect of Different Thawing Methods on the Physicochemical Properties and Microstructure of Frozen Instant Sea Cucumber

**DOI:** 10.3390/foods11172616

**Published:** 2022-08-29

**Authors:** Xiaotong Ge, Hongli Wang, Mingyu Yin, Xichang Wang

**Affiliations:** College of Food Science and Technology, Shanghai Ocean University, Shanghai 201306, China

**Keywords:** frozen instant sea cucumber thawing methods, water retention, mechanical properties, protein properties, microstructure

## Abstract

To provide recommendations to users regarding which thawing method for frozen instant sea cucumbers entails lower quality losses, in this study we compared the water retention, mechanical properties, protein properties, and microstructures of frozen instant sea cucumbers post-thawing by means of different thawing approaches, including refrigerator thawing (RT), air thawing (AT), water immersion thawing (WT), and ultrasound-assisted thawing (UT). The results indicated that UT took the shortest time. RT samples exhibited the best water-holding capacity, hardness and rheological properties, followed by UT samples. The α-helix and surface hydrophobicity of WT and AT samples were significantly lower than those of the first two methods (*p* < 0.05). The lowest protein maximum denaturation temperature (Tmax) was obtained by means of WT. AT samples had the lowest maximum fluorescence emission wavelength (λmax). Based on these results, WT and AT were more prone to the degradation of protein thermal stability and the destruction of the protein structure. Similarly, more crimping and fractures of the samples after WT and AT were observed in the sea cucumbers’ microstructures. Overall, we observed that UT can be used to maintain the quality of frozen instant sea cucumbers in the shortest time.

## 1. Introduction

Sea cucumber (*Stichopus japonicus*) has become an increasingly popular marine food in recent years in many eastern Asian countries and a few European countries, including China, Japan, and Russia, because of its nutritive value and rich bioactive components such as peptides, saponins, and alkaloids [1]. Currently, the annual production of sea cucumber reaches around 220,000 tons, with an output value of more than 7 billion U.S. dollars [2]. Sea cucumber is well-known to automatically decompose following various stimulations, such as mechanical damage, temperature changes, water quality changes, and salinity changes [3]. Autolysis is a physiological reaction to the degradation of the dermis (body wall) due to protein digestion, resulting in a serious degradation in the quality and economic value of the sea cucumber after harvest [4]. Therefore, it is of great significance to adopt an optimal processing method for storing fresh sea cucumbers after capture. Solar drying and pickling using a large amount of sugar or salt is the conventional processing method for sea cucumber [5]. However, this procedure is complicated and requires rehydration operations, which greatly degrades the intrinsic qualities of sea cucumbers. Unlike conventional dried sea cucumbers, instant sea cucumbers processed at high temperature and high pressure have become more and more popular because of their convenience, favorable textural properties, and minimal loss of nutrients.

Instant sea cucumbers are usually stored frozen, owing to their high moisture content of up to 92.59% [6]. Thawing, which is the opposite process to freezing, is indispensable before consuming the frozen instant sea cucumbers, and in this process the aim is to maintain the original food quality as much as possible. The methods of refrigerator (RT), air (AT), and water thawing (WT) are usually adopted for food thawing [7]. Refrigerator thaw is conducive to reducing losses of food quality during production [8]. Air thawing is one of the simplest thawing methods, which is based on the condition that the air temperature is higher than that of the frozen product, thus transferring heat to the frozen product to defrost it [9]. Water immersion thawing is a simple, easy and inexpensive method of thawing frozen products by placing them in a container filled with water, which absorbs heat. Choi et al. [10] found that thawing frozen pork loins with low-temperature water helped to restore its original hardness. Recently, a method of ultrasound-assisted (UT) [11] has gained popularity because of the characteristics of low quality losses and high efficiency. The ultrasonic method converts acoustic energy to thermal energy through the rupture of cavitation bubbles, and thermal energy speeds up the speed of the phase transition from ice to water, which shortens thawing time and makes frozen food thaw more uniformly. Wang et al. [12] noted that 25 °C ultrasonic stream hydrolysis (200 W) effectively slowed down the degree of oxidation of protein and fat, which maintained the quality of mackerel. Gambuteanu et al. [13] consistently found a significant reduction in thawing time using a 25 kHz ultrasound method, in which the mechanical properties of pork muscle were not significantly negatively affected.

The currently available literature has mainly focused on the impact of processing and freeze-thawing on the quality of frozen instant sea cucumbers [14,15], and the influence of different thawing methods has rarely been studied. The choice of thawing methods had a potential impact on the quality of the food. Therefore, in this study we aimed to compare the effects of four thawing methods (RT, AT, WT, and UT) on the water retention, moisture migration, mechanical properties, protein properties, and microstructure of frozen instant sea cucumbers.

## 2. Materials and Methods

### 2.1. Sample Preparation

Frozen instant sea cucumbers (Blue Bay Marine Food Co., Ltd., Dalian, China) (weight: 20.56 ± 1.78 g, body length: 8.12 ± 0.67 cm, *n* = 48) were purchased from a Metro Supermarket (Shanghai, China) and were stored at −20 °C ± 1 °C until use. A fresh-keeping box covered with ice packs was used for midway transportation. Four thawing methods were performed, namely, refrigerator thawing (RT, 4 °C ± 1 °C), air thawing (AT, 20 °C ± 2 °C), water immersion thawing (WT, water temperature 20 °C ± 1 °C) and ultrasonic-assisted thawing (UT, water temperature 20 °C ± 1 °C). UT was performed in an ultrasonic cleaner (KQ-100VDE, Kunshan Ultrasonic Instrument Co., Ltd., Kunshan, China) with a frequency of 43 kHz and a power of 200 W. Thawing curves were recorded by means of a temperature recorder (Fluke-NetDAQ32, Fluke electronic instrument Co., Ltd., Washington, D.C, USA) and thawing time was recorded when the center temperature reached 4 °C.

### 2.2. Determination of Water Retention and Moisture Migration

#### 2.2.1. Thawing Loss

The thawing loss of the instant sea cucumbers was determined based on the known weight of samples before (m_1_, g) and after (m_2_, g) thawing. It was calculated using the following formula:

(1) $$\mathrm{Thawing}\;\mathrm{loss}(\%)=(\frac{m_{1}-m_{2}}{m_{1}})\times 100$$

#### 2.2.2. Cooking Loss

Thawed instant sea cucumbers (5.00 ± 0.05 g) were weighed (m_3_, g) and placed in polyethylene bags. Next, the samples were placed in a water bath at 85 °C for 25 min and then cooled to room temperature, dried to the surface and weighed (m_4_, g) [16]. The cooking loss was obtained according to the formula as follows:

(2) $$\mathrm{Cooking}\;\mathrm{loss}(\%)=(\frac{m_{3}-m_{4}}{m_{3}})\times 100$$

#### 2.2.3. Water-Holding Capacity (WHC)

Thawed instant sea cucumbers (5.00 ± 0.05 g) (m_5_, g) were wrapped using filter papers with two layers and were transferred into a 50 mL centrifuge tube. They were centrifuged at 4 °C with a speed of 5000 r/min for 10 min. The samples were removed by peeling off the filter paper and the post-centrifugal mass was determined (m_6_,g) [12]. The calculation method of WHC was as follows:

(3) $$\mathrm{WHC}(\%)=(\frac{m_{6}}{m_{5}})\times 100$$

#### 2.2.4. Moisture Migration

Moisture migration was measured using a nuclear magnetic resonance (NMR, Suzhou Neuman Analytical Instrument Co., Suzhou, China). After thawing, the samples were cut into small pieces with the specification of 2.0 cm × 2.0 cm × 2.0 cm before being transferred into analysis tubes. The transverse relaxation time (T_2_) was determined based on the Carr–Purcell–Meiboom–Gill (CPMG) sequence. Parameters were set as follows: SF = 21 MHz, P_1_ = 13 μs, P_2_ = 26 μs, TW = 3000 ms, SW = 100 KHz, RFD = 0.080 ms, RG_1_ = 20.0 db, DRG_1_ = 1, PRG = 1, NS = 8, TE = 0.3 ms, NECH = 3000 [2].

### 2.3. Determination of Mechanical Properties

#### 2.3.1. Texture Profile Analysis (TPA)

The TPA was performed according to the methods reported previously with a minor modification [17]. Cylindrical samples were cut from the back of instant sea cucumbers with 2 cm diameter and 25 mm thickness and were transferred to a texture analyzer machine (TA-XT2I, Stable Micro Systems, London, UK) equipped with a P/50 probe. The testing speed was set as 1.0 mm/s with a 5.0 g trigger force point. Each sample was compressed twice at a degree of 30% and 5 s intervals.

#### 2.3.2. Rheological Properties

The rheological properties of samples were evaluated referring to previous methods [18], using an MCR301 rheometer (Anton Paar, Graz, Austria). The thawed samples were chopped and for the rheometer we used a 40 mm diameter aluminum plate. The samples were placed flat and dispersed, and the distance between the plate and the platform was set at 1000 μm. After loading, the samples were rested for 1 min at 25 °C and the device was sealed with liquid silicone oil to prevent moisture evaporation. A small-amplitude oscillatory test was undertaken to evaluate the dynamic viscoelastic properties with an increasing frequency from 1 to 100 rad/s. The storage modulus (G′) and loss modulus (G″) were recorded.

### 2.4. Determination of Protein Properties

Extraction of body wall proteins: frozen instant sea cucumbers thawed in different ways were added to lysis solution (containing 20 mmol/L pH 7.4 Tris-HCl, 2 mmol/L EDTA-2Na, 150 mmol/L NaCl, 10 mmol/L DTT, 1% Triton X-100, 0.1% SDS) (Maclean Biochemical Technology, Shanghai, China) at a ratio of 1:3 (*m*:*v*) in an ice bath. The proteins were extracted by grinding in an ice bath and then centrifuged at 4 °C for 15 min at 8000 r/min. The supernatant was freeze-dried and stored in portions [2].

#### 2.4.1. Protein Thermal Stability

A crucible with 4–8 mg protein freeze-dried powder was prepared. An empty crucible was used as a blank sample. For the measurement of maximum denaturation temperature (Tmax), we used a micro-SC differential scanning calorimeter (DSC) (PerkinElmer Instrumentation, Shelton, CT, USA). We cooled the samples to 20 °C and heated them at a speed of 5 °C/min until the temperature reached 150 °C [19].

#### 2.4.2. Protein Secondary Structure

For the characterization of functional groups in proteins, Fourier transform infrared radiation (FTIR) scans within 4000–1000 cm^−1^ of protein freeze-dried powder samples were analyzed using an attenuated total reflectance (ATR) cell [20]. Spectra were represented in Spectrum 10 STD software (PerkinElmer Instrumentation, Shelton, CT, USA). The secondary structure of proteins was calculated using Peakfit Version 4.12 software (Systat Software Inc., Palo Alto, CA, USA) with the algorithm method of Gaussian peak fitting. The contents of the secondary structure were obtained based on the relative areas of specific bands within the region of amide I (1700–1600 cm^−1^) [21].

#### 2.4.3. Protein Tertiary Structure

The protein tertiary structure (0.1 mg/mL) of the freeze-dried powder sample was measured using an F-7100 fluorescence spectropolarimeter (Hitachi Co., Tokyo, Japan) with an excitation wavelength of 290 nm, a slit width of 5 nm, and an emission wavelength of 310–400 nm [22].

#### 2.4.4. Surface Hydrophobicity

The surface hydrophobicity was measured according to the method of a previous study [23]. Phosphate buffer (0.01 mol/L, pH 7.0) was used to dilute samples into different concentrations consisting of 0.2, 0.4, 0.6, 0.8, and 1.0 mg/mL. A volume of 20 μL, 8 mmol/L ANS was added to a 4 mL protein solution with a 10 min reaction time in the dark. The value of optical density was measured with a 390 nm excitation wavelength and a 470 nm emission wavelength using an F-7100 fluorescence spectrophotometer (Hitachi Co., Tokyo, Japan). The linear relationship between fluorescence intensity and protein concentration was calculated, and the slope (k) was obtained, which was used as the surface hydrophobicity index of the protein molecule (H_0_).

### 2.5. Determination of Scanning Electron Microscopy (SEM)

The tissue structure of thawed instant sea cucumbers was photographed by means of SEM according to the method of Liu et al. [24]. The block of samples (1.0 cm × 1.0 cm × 1.0 cm) was fixed in a 2.5% glutaraldehyde solution at the temperature of 4 °C for 3 h and eluted with phosphate buffer (0.1 mol/L, pH 7.4) three times. The eluted samples were dehydrated, lyophilized, and sputtered with gold at 10 mA for 60 s, followed by observation in a SU8010 scanning electron microscope (Tokyo Hitachi Co., Ltd., Tokyo, Japan) at a 5 kV acceleration voltage and 800× magnification.

### 2.6. Principal Component Analysis (PCA)

Use the plug-in XLSTAT (Addinsoft Corporation, New York, NY, USA) in Excel for analysis, we imported the data and selected the PCA command. We selected the observations/variables in the data format field. We selected correlation in the PCA type field. The PCA type that was used during the computations was the correlation matrix, which corresponds to the Pearson correlation coefficient.

### 2.7. Statistical Analysis

All experiments were performed at least three times. Data were presented as the mean ± standard deviation (SD) obtained from the one-way analysis of variance (ANOVA) using SPSS 16.0 software (SPSS Inc., Chicago, IL, USA). Plotting was performed using Sigma Plot 12.5 (Systat Inc., San Jose, CA, USA).

## 3. Results and Discussions

### 3.1. Thawing Curve

**Figure 1:**
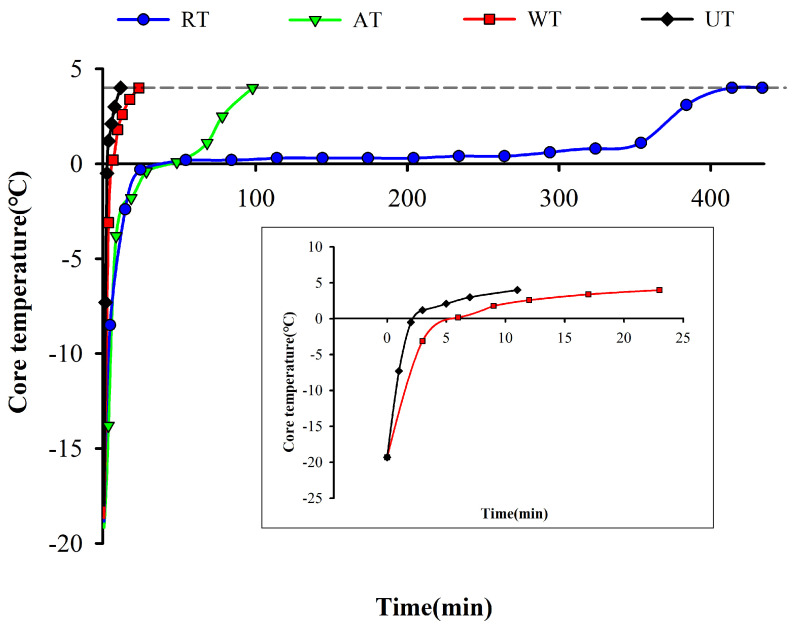
The time-temperature curves of instant sea cucumbers subjected to four thawing methods. In the gray box, we present the thawing curves for WT and UT from 0 to 25 min. RT, refrigerator thawing; AT, air thawing; WT, water immersion thawing; UT, ultrasonic-assisted thawing. The abbreviations of thawing methods are the same below.

As shown in Figure 1, the thawing times for RT, AT, WT, and UT were 414.9, 98.6, 23.1, and 11.3 min, respectively. The slope of RT and AT decreased from −5 °C to 0 °C, the thawing curve flattened out, and the thawing rate was low. This stage was the maximum ice crystal dissolution zone, when a large number of ice crystals began to melt. This process required more heat due to the phase change that occurred. As the ice decreased, the thermal conductivity of the sample decreases, so the thawing rate gradually decreased and thawing was slow [25]. On the other hand, WT accelerated the heat transduction due to water acting as a medium. UT required a shorter time than the other three methods. On the one hand, the micro-jet and cavitation effects generated by ultrasound during propagation can significantly increase the heat transfer coefficient. On the other hand, the attenuated acoustic energy is absorbed by the tissues of the instant sea cucumber and converted into heat, resulting in simultaneous internal and external thawing and accelerating the thawing rate [26]. Miles et al. [27] found that ultrasound leads to a greater attenuation in the frozen zone than in the unfrozen zone and that the attenuation increases significantly with temperature, reaching a maximum at the initial freezing point, thus allowing ultrasonic thawing to pass rapidly through the zone of maximum ice crystal production.

### 3.2. Water Retention and Moisture Migration

#### 3.2.1. Water Retention

In the process of thawing, water within samples was changed from solid ice to liquid water. Most crystalline water was absorbed again at the beginning of thawing. A portion of the total water leaked out through the extracellular matrix, resulting in liquid losses [28,29]. As shown in Table 1, the thawing losses for RT samples were significantly less than those of the other thawing methods (*p* < 0.05). The water-holding capacity for RT samples was 4.03%, 5.31%, and 9.45% higher than those of UT, HT, and AT samples, which is similar to the results of a study on quality losses in mackerel caused by different thawing methods [12], in that RT performed relatively well in retaining water during thawing. As RT possesses a lower temperature, the milder thawing process might reduce the intensity of biochemical reactions and inhibit the reproduction of microorganisms, which facilitates the reabsorption of water melted by ice crystals in the interstitial space into the cells, thereby reducing thawing losses and increasing water-holding capacity [30]. With the assistance of ultrasound, UT could pass the maximum ice crystal melting zone from −5 °C to 0 °C more rapidly with a decrease in protein hydration and an increase in water retention.

#### 3.2.2. Moisture Migration

**Figure 2:**
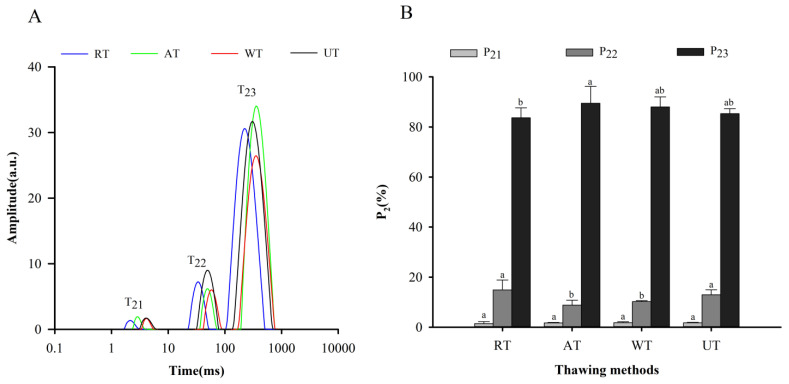
Influence of different thawing methods on the T_2_ relaxation time (**A**) and relative water content (**B**) of frozen instant sea cucumber. Values of different groups with different lowercase letters (a–b) are significantly different at *p* < 0.05.

The relaxation time (T_2_) reflects the mobility and distribution of water molecules in post-thawing instant sea cucumbers through the level of hydrogen proton confinement [31]. The bound water, immobile water, and free water were concentrated in 1–10 ms (T_21_), 10–100 ms (T_22_), and 100–1000 ms (T_23_), respectively (Figure 2A). The highest relaxation time was detected in WT samples, followed by AT, UT, and RT samples. The scale of the relaxation time was proportional to the strength of free water fluidity [32]. Therefore, WT may have higher free water fluidity than others. As for the reasons behind this, thawing is a process of heating and oxidation. The degree of thawing-induced oxidation is related to water fluidity, as Sun et al. [33] suggested that oxidation exposes the inner hydrophobic groupings of proteins and decreases the binding of proteins to water, which ultimately increases the degree of freedom of water.

Figure 2B represents the area proportions of various water peaks for instant sea cucumber processed using four thawing methods. Comparing the area proportions of various water types, bound water (P_21_) occupied the smallest area at 2%, immobile water (P_22_) occupied about 12%, and free water (P_23_) reached almost 86%. Liu et al. [2] also found that the calculated peak areas of bound water, immobile water, and free water were 91.83 ± 2.80, 179.42 ± 1.71, and 2493.20 ± 2.14 g^−1^ for instant sea cucumbers, which indicates that free water content greatly outnumbered bound water and immobile water. After cooking, proteins of the instant sea cucumber body wall were depolymerized with enlarged gaps between fibers, leading to an increase in free water mobility [15]. In addition, the difference in bound water among different thawing methods was not significant (*p* > 0.05). This result may be because bound water was tightly bound to the proteins of the sea cucumber body wall, independent of any mechanical stress or microstructural changes, and could withstand the temperature increase during the thawing process [34]. The ranking trend of P_22_ was opposite to that of P_23_, due to the fact that the water population in the sample processed using different thawing methods was able to generate mutual transformation [35]. P_22_ was significantly higher in RT and UT samples than in AT and WT samples (*p* < 0.05), suggesting that RT and UT samples could retain more intracellular water.

### 3.3. Mechanical Properties

#### 3.3.1. TPA

**Figure 3:**
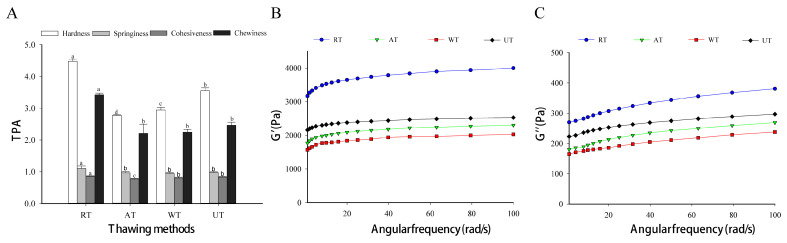
Influence of different thawing methods on TPA (**A**) and rheological properties (**B**,**C**) of frozen instant sea cucumber. (**B**,**C**) represent changes in the storage modulus and loss modulus. The units of hardness and chewiness are kilograms (kg). Values of different groups with different lowercase letters (a–d) are significantly different at *p* < 0.05.

TPA is an empirical method that has been extensively applied to the analysis of food mechanical properties in terms of deformation for texture assessments [36,37]. The hardness, springiness, cohesiveness, and chewiness results of thawed instant sea cucumber are listed in Figure 3A. RT samples had the highest hardness values (4.48 kg) followed by UT (3.55 kg) and both thawing methods exhibited significantly higher hardness values than AT and WT (*p* < 0.05). This may be related to the fact that RT had a lower thawing temperature and UT had a faster thawing rate. High hardness indicates a close aggregation of proteins and a relatively dense structure of the fiber network, maintaining the original shape. A similar trend was observed in chewiness. The chewiness value of RT samples (3.43 kg) was significantly greater than those of the other thawing methods (*p* < 0.05). For springiness, a non-significant difference was seen among AT, WT, and UT samples (*p* > 0.05), which might be attributed to the low fat content in frozen instant sea cucumbers and the short thawing time, which does not cause significant fat loss. Wu et al. [38] found that the change in food springiness was positively correlated with fat content. The cohesiveness, which was closely related to samples’ flexibility and their ability to resist tissue damage, referred to the degree of deformation before sample breakage [12].

#### 3.3.2. Rheological Properties

The instant sea cucumber is a typical complex with the properties of liquid viscosity and solid springiness. The index G′ is related to sample springiness, continuously ascending with an increase in angular velocity [19,39]. Figure 3B indicates the significant changes in G′ in RT samples (*p* < 0.05). The index increased by 1600 Pa at 1 rad/s and 1970 Pa at 100 rad/s, compared with WT samples. Previous studies have suggested that a decrease in G′ may indicate a reduction in mechanical strength caused by the uncoupling of protein triple helices to random coils [3].

The loss modulus G″ is used to evaluate sample viscosity [18]. As shown in Figure 3C, the loss moduli and storage moduli of the four thawing methods exhibited the same trends. The loss modulus indirectly reflected sample viscosity, which was closely associated with protein conformation and aggregation structure. In addition, all G′ values were about 10 times the corresponding G″. Both of them climbed slowly with the increase in the angular frequency, which might be explained by the cross-linkage formed by disulfide bonds based on the principle of hydrophobic interactions [40].

### 3.4. Protein Properties

#### 3.4.1. Thermal Stability of Proteins

**Figure 4:**
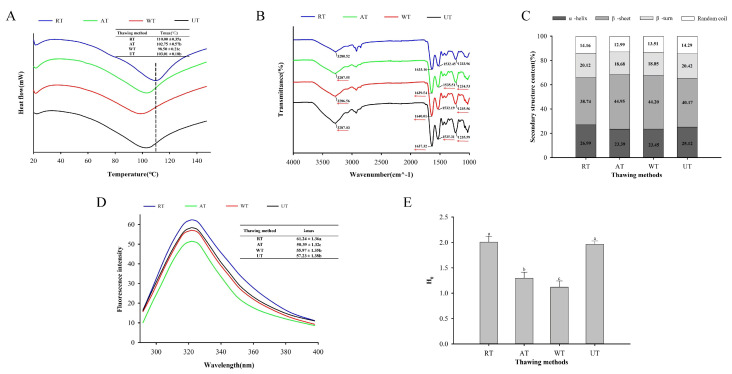
Influence of different thawing methods on the differential scanning calorimetry (DSC) (**A**), fourier transform infrared radiation (FTIR) (**B**), protein secondary structure (**C**), fluorescence spectroscopy (**D**), and surface hydrophobicity (**E**) characteristics of frozen instant sea cucumber. Values of different groups with different lowercase letters (a–c) are significantly different at *p* < 0.05.

Differential scanning calorimetry (DSC) relates to variations in protein structure [41]. DSC thermograms of frozen instant sea cucumbers post-thawing by means of four different methods are shown in Figure 4A, in which a principal peak can be observed. The maximum denaturation temperature (Tmax) of RT samples was 110.00 °C ± 0.35 °C, which was close to the previously reported measurement of 103.9 °C [42]. With the unchanged DSC temperature spectrum, the Tmax of RT samples significantly surpassed those of the AT and UT samples (*p* < 0.05), and the latter were also significantly higher than WT samples (*p* < 0.05). Decreased Tmax suggested damage to the secondary or tertiary protein structures caused by heating or oxidation. The triple helix structure was untied or broken into smaller molecular chains, with a gradually reduced stability in response to heat [24].

#### 3.4.2. Secondary Protein Structure

The infrared spectra of frozen instant sea cucumbers at the wavelength of 1000–4000 cm^−1^ were measured by means of Fourier-transform infrared spectroscopy, wit hthe aim of evaluating the changes in protein conformation. Functional groups of collagen were mainly distributed at the wavenumbers of 3300 cm^−1^ (amide A), 1640 cm^−1^ (amide I), 1535 cm^−1^ (amide II), and 1235 cm^−1^ (amide III) [23]. As shown in Figure 4B, there was an apparent absorption peak in the main wave number, indicating that collagen was the main protein in instant sea cucumbers. In addition, based on the RT samples, the amide bands A, Im and III in other thawing methods demonstrated redshifts, and the amide band II in AT and UT samples showed redshifts. Higher wavenumbers of amide bands indicate low protein structural stability and weakened hydrogen bonds [43]. The amide A band consisted of N-H stretching vibrations [44]. The amide I band was generated by a carbonyl (C=O) stretching vibration, which provided an important clue for the analysis of the secondary protein structure. Amide II was associated with the N-H bond and C-N expansion, and amide III was related to C-N stretching and N-H bending, which take part in the triple helix structure of the protein [45]. These results indicate that different thawing methods may impact protein structural stability by changing the protein conformation, through processes such as C=O stretching, N-H stretching, N-H bending, and C-N stretching.

The secondary structure of proteins might be assessed according to amide I (1600–1700 cm^−1^) via infrared spectroscopy because C=O forms hydrogen bonds with neighboring groups [46]. Gaussian fitting and relative percentage calculation results are shown in Figure 4C. The α-helix content of RT samples was higher than that of the other methods, even though the changing direction of the β-sheet was opposite to the α-helix, which was consistent with previous findings [47]. For AT and WT samples, there were more structural transitions from the α-helix to the β-sheet, and the rearrangement of hydrogen bonds in proteins weakened the stability of the protein structure, which might be attributed to the exposure of hydrophobic regions induced by thawing methods and the subsequent intramolecular reduction and breakage of hydrogen bonds [33]. The number of β-turns in UT samples outnumbered those of AT and WT samples. The disparities in the secondary protein structures of instant sea cucumbers after thawing can be explained by the unfolding, depolymerization, and rearrangement of protein molecules due to the production of free radicals induced by thawing, which leads to the breaking of covalent bonds (including peptide bonds) [48], ultimately causing significant changes in mechanical properties. These results are consistent with the changes in mechanical properties described in Section 3.3.

#### 3.4.3. Tertiary Protein Structure

Fluorescence spectroscopy is capable of tracking the transitions of the tertiary protein structure during food processing and storage. Tryptophan (Trp) and other hydrophobic amino acid residues leave the core of the protein following damage to the protein tertiary structure, leading to a drop in fluorescence intensity [8]. As shown in Figure 4D, the maximum fluorescence emission wavelength (λmax) of all samples was close to 320 nm. RT samples had the highest fluorescence intensity, which might be due to a low-temperature environment during RT processing, along with the ameliorated oxidation of the protein structure. The fluorescence intensities of UT and WT samples were both lower than those of RT samples, with a non-significant difference in the fluorescence intensity between UT and WT samples (*p* > 0.05), which indicated that UT or WT accelerated thawing and retarded the oxidation of instant sea cucumber proteins. The fluorescence intensity of AT samples was significantly lower than that of the others (*p* < 0.05). This decrease in fluorescence intensity may be due to fluorescence quenching caused by the recombination or aggregation of hydrophobic groups [49]. Zhang et al. [50] reported that the decrease in fluorescence intensity is caused by protein refolding, which leads to the returning of exposed Trp residues to the protein interior.

#### 3.4.4. Surface Hydrophobicity

Surface hydrophobicity (H_0_) is the primary force involved in maintaining the tertiary structure of proteins, which is imperative for the stability and functionality of protein structure [8,18]. Figure 4E depicts the influence of various thawing approaches on the surface hydrophobicity of instant sea cucumbers. The surface hydrophobicity of RT and UT samples was significantly higher than that of WT and AT samples (*p* < 0.05), which suggested that RT might enhance the unfolding of protein molecules in instant sea cucumbers and cause the exposure of hydrophobic groups from the inner side to the outer surface, thereby increasing the surface hydrophobicity [51]. The decrease in surface hydrophobicity of WT and AT samples might be attributed to hydrophobicity-induced protein proximity and aggregation, thus wrapping exposed hydrophobic groups in proteins [52].

### 3.5. Microstructure

**Figure 5:**
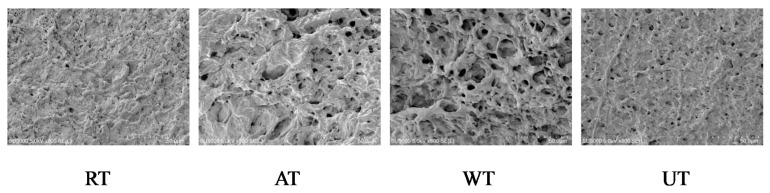
Influence of different thawing methods on the microstructures of frozen instant sea cucumbers (magnification: 800×).

The impact of diverse thawing methods on the microstructures of instant sea cucumbers is shown in Figure 5. Changes in moisture migration and protein oxidation during the thawing process led to destruction of the protein structure, which directly affected the microstructure [53]. As described in Figure 5, RT samples presented many uneven fragments, gaps, and pores of small size. In contrast with RT samples, increased numbers of pores with smaller and more uniform shapes were observed in UT samples. In AT and WT samples, we could observe more crimping, fractures and denaturation, increased numbers of pores with an uneven distribution, and fractured fibers encapsulating the pores, which loosened the sample texture. The results indicated that RT caused minimal disruption to the microstructure, followed by UT, WT, and AT. This outcome is similar to the findings of Xia et al. [54], who found that RT samples presented the least damage to the microstructure of the porcine longissimus muscle.

### 3.6. Principal Component Analysis

**Figure 6:**
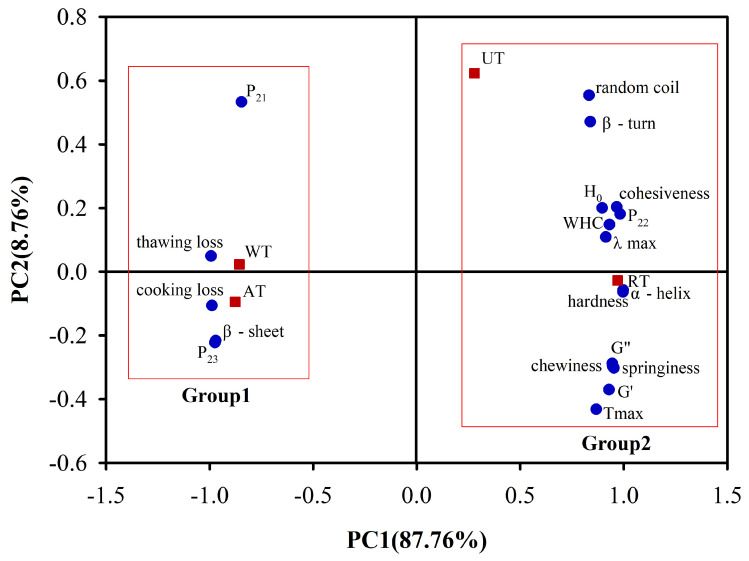
Principal component analysis (PCA) of water retention, mechanical properties, and protein properties of instant sea cucumbers thawed with different thawing methods.

In the above analyses, we observed similar trends in terms of the water retention, mechanical properties, and protein properties of sea cucumber samples processed using four thawing methods. Therefore, we used PCA to analyze the linear combination of the moisture, mechanical, and protein properties of instant sea cucumber post-thawing. As shown in Figure 6, the PCA results revealed that the first two principal components (PCs) were responsible for 96.52% of the total variance of the data (87.76% PC1 and 8.76% PC2), which indicated there was a strong correlation between the selection of thawing methods and the quality of frozen instant sea cucumber. The physicochemical properties and thawing methods were located in quadrants 1, 2, 3, and 4. On the other hand, the treatments located in quadrants 1, 2, 3, and 4 were related to the variables in the corresponding quadrants [55]. The four thawing methods can be divided into two groups based on the horizontal coordinate PC1, i.e., we observed a clear difference between these two groups of frozen instant sea cucumbers. The first group is characterized by AT and WT samples, as these were the samples with the highest thawing loss, cooking loss, P_21_, P_23_, and β-sheets. The second group includes RT and UT samples, with the highest WHC, P_22_, TPA, G′, G″, Tmax, α-helix, β-turn, random coil, λmax, and H_0_ values. Based on these results, we inferred that the frozen instant sea cucumber samples obtained after RT and UT were of better quality than those obtained after AT and WT. Furthermore, the α-helix was very closely linked to hardness, Tmax to the loss modulus G′, and H_0_ to cohesiveness. We hypothesized that the destruction of the protein structure was primarily responsible for the decrease in the mechanical properties of instant sea cucumbers after thawing.

### 3.7. Limitations

A limitation of this study is that the differences in quality compared to fresh sea cucumbers before freezing were not studied. This is limiting because the difference in quality after thawing may also be due to individual differences in the sea cucumbers themselves. Furthermore, because the frozen instant sea cucumber products purchased for thi study were commercially available, there were differences in the freezing processes used in different factories, which could also have an impact on the quality of the sea cucumbers. Therefore, in future studies, researchers could consider purchasing fresh sea cucumbers and observing the changes in quality before freezing and after thawing. However, because of the extremely autolytic nature of sea cucumbers, quality evaluations of fresh sea cucumber before freezing may lead to changes in sea cucumber quality and introduce confounding factors. Therefore, in this study we selected frozen instant sea cucumber products of the same brand, with the same processing location, the same release date, and similar sizes to those sold in the market. It is worthwhile to give more thought to the question of how to better control the experimental variables when conducting future studies on variations in the quality of frozen instant sea cucumbers.

## 4. Conclusions

In this study we aimed to assess the effects of four thawing methods on the physicochemical properties and microstructures of frozen instant sea cucumbers. There were significant differences in water retention, mechanical properties, protein properties, and microstructures between the four selected thawing methods. Among them, the samples obtained after RT exhibited the highest quality, along with the longest thawing time. UT was second to RT, but with a noticeable reduction in thawing time, and mostly performed better than AT and WT, especially in terms of hardness. However, UT exhibited non-significant differences compared with WT in terms of the water-holding capacity, springiness, cohesiveness, chewiness, and λmax values (*p* > 0.05). Compared to the two abovementioned methods, AT and WT exhibited lower post-thawing sample quality, in that they demonstrated significantly reduced food hardness, G′, G″, WHC, Tmax, α-helix, λmax, and surface hydrophobicity values, with an increase in thawing losses, cooking losses, relative free water content, β-sheets, and pore size. PCA analysis also strengthened these findings. Briefly, UT was found to be a time-saving thawing method for frozen instant sea cucumbers, showing the best performance in maintaining food quality among the four thawing methods. The selection of UT for the thawing of frozen instant sea cucumber can better satisfy industrial requirements and provide actual usage guidance to consumers after sale.

## Figures and Tables

**Table 1:** Influence of thawing methods on the thawing loss, cooking loss, and water-holding capacity of frozen instant sea cucumber.

Thawing Methods	Thawing Loss (%)	Cooking Loss (%)	Water-Holding Capacity (%)
RT	17.11 ± 1.30 c	9.59 ± 1.03 b	74.87 ± 0.60 a
AT	22.68 ± 1.00 a	13.44 ± 0.64 a	65.42 ± 2.82 c
WT	21.81 ± 1.73 ab	13.24 ± 0.50 a	69.56 ± 1.52 b
UT	20.15 ± 0.73 b	10.90 ± 0.00 b	70.84 ± 0.88 b

Note: Values of different groups with different lowercase letters (a–c) are significantly different at *p* < 0.05. RT, refrigerator thawing; AT, air thawing; WT, water immersion thawing; UT, ultrasonic-assisted thawing.

## Data Availability

The data presented in this study are contained within the article.
